# Potential Geographic Distribution and Suitable Area of Three Species of *Diabrotica* (Coleoptera: Chrysomelidae) Beetles in Corn-Planting Regions of China

**DOI:** 10.3390/insects16101072

**Published:** 2025-10-20

**Authors:** Yening Jin, Fan Shao, Sizhu Zheng, Yumeng Wang, Gao Hu, Fajun Chen

**Affiliations:** 1 State Key Laboratory of Agricultural and Forestry Biosecurity, College of Plant Protection, Nanjing Agricultural University, Nanjing 210095, China; 2024802145@stu.njau.edu.cn (Y.J.); yumengwang@njau.edu.cn (Y.W.); 2 Ji’an City Forest Pest Control and Quarantine Station, Jian 343000, China; shaofan187@163.com; 3 Animal, Plant and Food Inspection Center of Nanjing Customs, Nanjing 210019, China; zhengsizhu@126.com

**Keywords:** invasive pests, *Diabrotica virgifera*, *Diabrotica undecimpunctata*, *Diabrotica barberi*, potential geographic distribution, suitable habitats, overlay analysis, corn-growing regions, global warming

## Abstract

**Simple Summary:**

The *Diabrotica* beetles of *D. virgifera virgifera*, *D. undecimpunctata*, and *D. barberi* are important insect pests of corn crops that natively occur in America and are mainly distributed between 35–49° N. In addition, they have spread rapidly to many countries in Europe over the past 70 years since 1955. Corn is also one of the main crops in China; its planting area is mainly distributed between 30–50° N, and these geographical latitudes are similar to those of America and Europe. Once they invade into China, it is very likely that they will spread, settle, and develop rapidly in China. So, there is an invasion risk because these *Diabrotica* beetles can be naturally or anthropogenically transmitted into China. In this study, the potential geographic distribution of these *Diabrotica* beetles based on their global distribution samples and relevant bioclimatic variables was estimated, and an overlay analysis was further carried out in combination with the actual distribution of corn-growing regions in China, in order to assess their potential invasion risks, especially in the corn-planting regions of China, and in order to service the early warning and management of invasive crop pests under global warming.

**Abstract:**

Corn rootworms of *Diabrotica virgifera virgifera* Le Conte, 1868; *Diabrotica undecimpunctata howardi* Barber, 1947, and *Diabrotica barberi* R.F. Smith & Lawrence, 1967 are important pests of corn crops that natively occur in America and have a potential risk of spreading into China through natural spreading or anthropogenic invasion. In this study, the potential geographic distribution and suitable area of these three *Diabrotica* species based on their global distribution samples and relevant bioclimatic variables were estimated, and an overlay analysis was further carried out in combination with the actual distribution of corn-growing regions, especially in China, in order to assess the potential invasion risks of these *Diabrotica* beetles, especially in the corn-planting regions of China. The results indicated that six bioclimatic variables (i.e., bio2 (mean diurnal range), bio4 (temperature seasonality), bio5 (max temperature of the warmest month), bio6 (min temperature of coldest month), bio13 (precipitation of wettest month), and bio14 (precipitation of driest month)) were selected for the analysis of the potential geographic distribution and suitable areas of these *Diabrotica* beetles. The suitable area ranges of *D. undecimpunctata* and *D. virgifera virgifera* are relatively large in China, i.e., 21.01–48.46° N and 74.01–131.26° E for *D. undecimpunctata* and 21.58–41.42° N and 78.71–124.43° E for *D. virgifera virgifera*, respectively, while *D. barberi* occupies only a small area in China, i.e., 34.21–46.81° N and 108.80–133.75° E. Based on the overlay analysis of the potential geographic distribution of these three *Diabrotica* species and the actual distribution of corn-growing regions in China, *D. undecimpunctata* and *D. virgifera virgifera* have the largest potential geographic distribution areas, totaling 2.618 × 10^7^ ha and 1.814 × 10^7^ ha in 22 and 20 provinces respectively, while *D. barberi* has the lowest potential geographic distribution area just in 8 provinces, totaling 44.37 × 10^4^ ha, indicating a low-suitability area. Moreover, under the four climate scenarios (i.e., SSP1_2.6, SSP2_4.5, SSP3_7.0, and SSP5_8.5) in the 2030s and 2050s, these *Diabrotica* beetles have the potential for sporadic increases or decreases surrounding the potential suitable areas under the current scenario. However, it is worth noting that the high-suitability areas of *D. undecimpunctata* and *D. virgifera virgifera* decreased, and their medium- and low-suitability areas increased accordingly. It is presumed that *Diabrotica* beetles, especially *D. virgifera virgifera* and *D. undecimpunctata*, have a high risk of potential invasion into China because there is a large potentially suitable area distribution for their possible occurrence in the maize-planting regions of China.

## 1. Introduction

Corn rootworms are important insect pests of corn crops that natively occur in Central America [1]. There are three key species of corn rootworms that cause damage mainly to corn crops, that is, the western corn rootworm *Diabrotica virgifera virgifera* Le Conte, 1868, the southern corn rootworm *Diabrotica undecimpunctata howardi* Barber, 1947, and the northern corn rootworm *Diabrotica barberi* R.F. Smith & Lawrence, 1967. *D. virgifera virgifera* and *D. barberi* generally overwinter as eggs, while *D. undecimpunctata* generally overwinters as adults. *D. virgifera virgifera* larvae mainly damage corn roots [2,3], and their adults mainly feed on leaves and pollen of corn crops and some gramineous plants [4]. *D. virgifera virgifera* has been listed as A1 and A2 quarantine by the European and Mediterranean Plant Protection Organization (EPPO). Since 1867, it has gradually expanded from Central America to North America, and it invaded Europe in the 1990s [4,5], indicating its excellent adapting ability to various management strategies and successful invasion into new regions [6]. The invasion of *D. virgifera virgifera* has caused billions of USD of economic losses annually in global corn production [6,7]. To date, corn rootworms of *D. virgifera virgifera*, *D. undecimpunctata*, and *D. barberi* have not been detected in China; however, there are risks of invasion into China for these *Diabrotica* (Coleoptera: Chrysomelidae) beetles because they can be transmitted by vehicles (e.g., ships and airplanes, etc.) over long distances, and their adults have strong flight capacities [8].

Species distribution models (SDMs) based on species distribution plots and climatic variables are powerful tools for describing where invasive pests may arrive, establish, spread, or cause harmful effects [9,10]. To date, SDMs have evolved from the BlOCLlM model to include widely used models, such as MaxEnt (i.e., Maximum Entropy Model), SRE (i.e., Slicewise Envelopment Analysis), GLM (i.e., Generalized Linear Model), GAM (i.e., Generalized Additive Model), GBM (i.e., Boosted Generalized Bins Model), CTA (i.e., Classification Tree Analysis), MARS (i.e., Multivariate Autoregressive Spline), ANNk (i.e., Artificial Neural Network), RF (i.e., Random Forest), and FDA (i.e., Flexible Discriminant Analysis) [11,12,13]. Guéguen et al. [14] developed the “biomod2” software package based on the R package (version 4.3.3), providing a platform for more convenient use of combinatorial models. In addition, SDMs have been widely proven to have better predictive performance in evaluating suitable areas for the establishment of global invasive species [15,16,17]. Jin et al. [8] estimated the global geographical distribution patterns and ecological niche overlap of three species of corn rootworms, *D. virgifera virgifera*, *D. undecimpunctata*, and *D. barberi*, although they did not consider the actual distribution of the host corn crop, which is a necessary condition for its successful colonization and for assessing the damage caused by these *Diabrotica* beetles.

In this study, the potential geographic distribution of *D. virgifera virgifera*, *D. undecimpunctata*, and *D. barberi* was assessed, and an overlay analysis was further carried out in combination with an assessment of the actual distribution of corn-growing regions in China in order to assess the potential invasion and spreading risks of these *Diabrotica* beetles in China and to service the early warning and management of invasive crop pests under global warming.

## 2. Materials and Methods

### 2.1. Data Resources

#### 2.1.1. Occurrence Data of Three Species of *Diabrotica* Beetles

**Figure 1:**
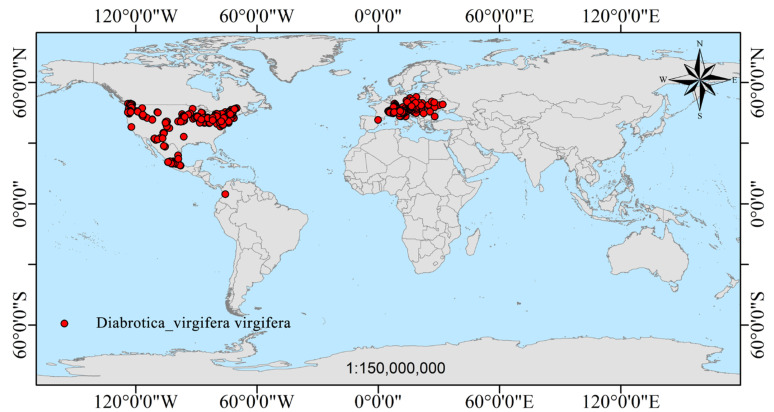

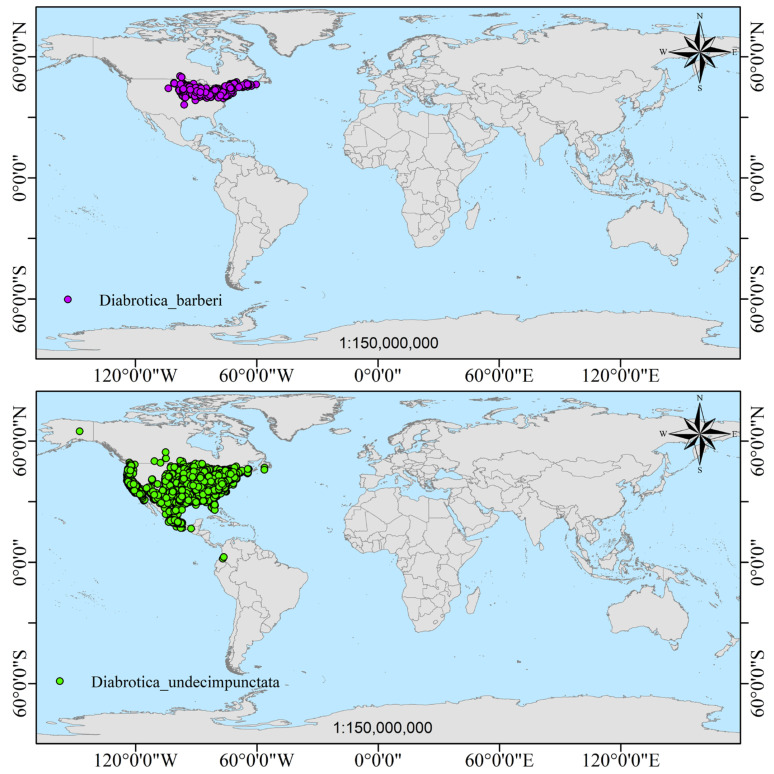
Global actual distribution maps of the three *Diabrotica* species, i.e., *D. virgifera virgifera*, *D. barberi*, and *D. undecimpunctata*.

The global occurrence data of the three species of corn rootworms, i.e., *D. virgifera virgifera*, *D. undecimpunctata*, and *D. barberi*, were obtained from GBIF (https://www.gbif.org/; accessed on 20 August 2024) and Web of Science (https://www.webofscience.com/; accessed on 20 August 2024). A total of 29,623 occurrence points were collected, including 27,705 for *D. undecimpunctata* (https://doi.org/10.15468/dl.4nh8ps), 1053 for *D. barberi* (https://doi.org/10.15468/dl.qzekfj), and 865 for *D. virgifera virgifera* (https://doi.org/10.15468/dl.7mwqss). The time range of all occurrence points was from 2000 to 2024. To reduce the impact of sampling bias on model development, the ‘spThin’ package (Version: 0.2.0) in the R software (https://cran.r-project.org/web/packages/spThin/index.html; accessed on 25 August 2024) was used to remove duplicate and erroneous occurrence points (Code S1). Occurrence data for each of the three *Diabrotica* species was filtered within a circle with a 5 km radius to ensure that only one occurrence point existed for each species within each circle. Finally, the MaxEnt model was run, considering 8235 occurrence points for *D. undecimpunctata*, 625 for *D. barberi*, and 547 for *D. virgifera virgifera* (Dataset S1), and the actual distribution of all these occurrence points of *D. virgifera virgifera*, *D. undecimpunctata* and *D. barberi* is shown in Figure 1, respectively.

#### 2.1.2. Climatic Data for Model Development

In order to align the time period of the environmental data with the occurrence period of the three *Diabrotica species*, i.e., *D. virgifera virgifera*, *D. undecimpunctata*, and *D. barberi*, this study utilized 19 bioclimatic variable factors from 2000 to 2024 for model development. These climate variable factors were generated through a procedural process as follows: Firstly, the monthly average maximum temperature, monthly average minimum temperature, and total precipitation data for each year from 2000 to 2024 with a spatial resolution of 2.5 min (about 5 km × 5 km) were obtained from the Worldclim database version 2.1. Then, using the R language version 4.3.3, the above data for each year were averaged to obtain 25-year average data for the monthly average maximum temperature, monthly average minimum temperature, and total precipitation (Code S1). Finally, using the method provided by WorldClim, the above three average data were processed with the ‘dismo’ package (Version: 1.3-16) [9], and the bioclimatic variable factors for the period from 2000 to 2024 were generated, as shown in Table 1, including bio2 (°C), bio4, bio5 (°C), bio6 (°C), bio13 (mm), bio14 (mm), and bio15 (mm).

In addition, four SSPs covering the periods of 2021–2040 and 2041–2060 were used to predict the future potential suitable habitats of these three species of *Diabrotica* beetles. These future data were extracted from the climate system model (BCC-CSM2-MR) of the Beijing Climate Center according to the CMIP6 protocol. This protocol provides global unified atmospheric predictions for niche modeling and is more suitable for studies related to habitat prediction under climate change in China [18].

#### 2.1.3. Data on China’s Corn-Planting Areas

**Figure 2:**
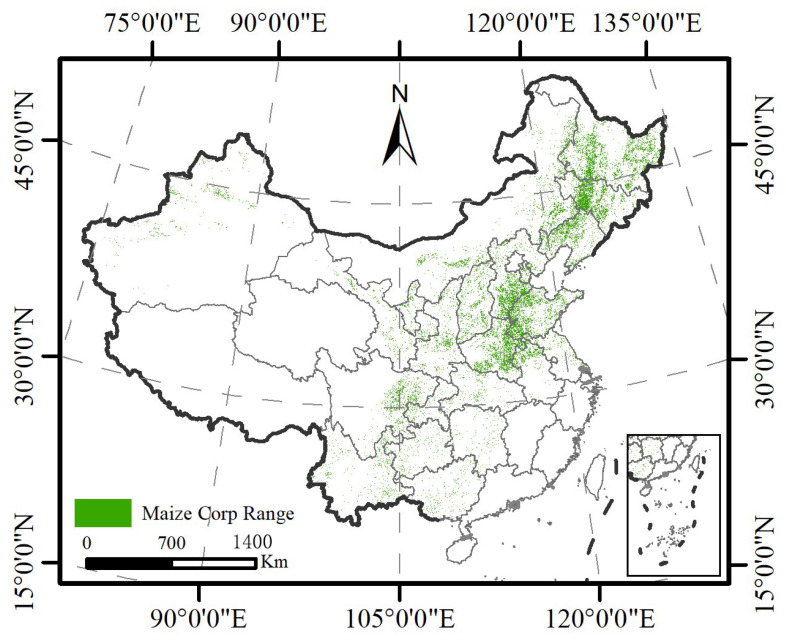
Geographic distribution of corn crop in China based on the downloaded data of China’s corn planting areas in 2023 from the National Science and Technology Infrastructure Platform—National Ecosystem Science Data Center (https://nesdc.org.cn/sdo/detail?id=651403fd7e281774b9b5da68; accessed on 21 August 2024).

Corn is the main host plant of the *Diabrotica* species and is a necessary condition for its successful colonization and damage. Therefore, the data on China’s corn-planting areas in 2023 was downloaded from the National Science and Technology Infrastructure Platform—National Ecosystem Science Data Center (https://nesdc.org.cn/sdo/detail?id=651403fd7e281774b9b5da68; accessed on 21 August 2024), with a resolution of 30 m by 30 m, and an actual national corn-planting distribution map of China in 2023 was created, which indicated that the national corn-planting area was as high as 44,218,940 hectares (Figure 2). Figure 2 also shows that the corn-planting region can be mainly divided into 5 major production areas as follows:, (1) The northern spring corn area, mainly consisting of the three northeastern provinces (including Heilongjiang, Jilin, and Liaoning) and Inner Mongolia, as well as some parts of Xinjiang. (2) The Yellow–Huaihai summer corn area, mainly including Hebei, Shandong, and Henan, including Beijing, Tianjin, Shanxi, Jiangsu, Anhui, etc. (3) The southwest mountainous corn area, mainly consisting of Sichuan, Guangxi, Yunnan, and Guizhou. (4) The northwest irrigation corn area, mainly including Xinjiang and Gansu, etc. (5) The southern hilly corn area, including Shanghai, Zhejiang, part of Anhui, Fujian, Jiangxi, Hubei, Hunan, Guangdong, Guangxi, and Hainan.

### 2.2. MaxEnt Model and Model Optimizing

MaxEnt has been widely proven to have better predictive performance in evaluating suitable areas for the establishment of global invasive species compared to other species distribution modeling methods [17]. Jin et al. [8] indicated that the variables bio2, bio4, bio5, bio6, bio13, bio14, and bio15 were the best factors for predicting the suitable areas of *D. virgifera virgifera*, *D. undecimpunctata*, and *D. barberi*. Therefore, these variables were used to predict the suitable areas of these three species of *Diabrotica* beetles. The number of background points was set to 10,000, and the random test percentage was setup as 25% whilst simultaneously selecting “random seed” for the test repeats. Here, the model was run 10 times, and the values were averaged.

In this study, the MaxEnt model mainly tuned two parameters, i.e., regularization multiplier (RM) and feature combination (FC). The RM and FC parameters were optimized using the R package ENMeval with “randomkfold” [19,20]. The 5 basic elements of the FC parameter were linear (L), quadratic (Q), hinge (H), product (P), and threshold (T) functions. By combining them, 11 feature combinations were generated, i.e., LQH, LQP, LQT, LHP, LHT, LPT, LQHP, LQHT, LHPT, LQPT, and LQHPT. The RM parameter was set to 0.5–4.0, with a step size of 0.5, and total of 88 adjusted and optimized combinations were generated. The complexity of the MaxEnt model was evaluated by modified Akaike information criterion (AIC) values under different parameter combinations. The delta Akaike information criterion (ΔAICc) was used to determine the parameters corresponding to the minimum value, which were the optimal parameters [21].

### 2.3. Model Evaluation and Habitat Suitability Classification

Model evaluation is an indispensable part of species-specific distribution modeling. Through rigorous evaluation, researchers can ensure the reliability, accuracy, and practicality of the model, thereby providing a solid foundation for species distribution research and environmental protection [22]. The commonly used indicator is AUC (i.e., area under the curve), usually referring to the area under the receiver operating characteristic curve (i.e., the ROC curve) [22]. When evaluating the performance of classification models, AUC is an important metric, with a value ranging from 0 to 1 (AUC = 1 indicates perfect prediction by the model; AUC = 0.5 indicates that the model has no predictive ability and performs as randomly guessing; AUC < 0.5 indicates that the model’s performance is worse than random guessing). The ROC curve is a graphical tool used to evaluate the performance of classification models [23], and it depicts the performance of the model at all possible classification thresholds, including the true positive rate (TPR), false positive rate (FPR), and true skill statistic (TSS). TSS considers the missing mean errors and is not affected by the size of the verification dataset. In addition, the ROC curve is plotted by using FPR as the x-axis and TPR as the y-axis. A value of AUC < 0.6 indicates unreliable model results, between 0.6 and 0.7 indicates poor model performance, between 0.7 and 0.8 indicates average model performance, between 0.8 and 0.9 indicates good model performance, and >0.9 indicates excellent model performance [24].

The classification of suitable habitat areas is an important step to study the potential suitable habitats of invasive species, and the suitability degree of species can be divided into four intuitive levels, i.e., highly suitable (indicating that the environmental conditions in these areas closely match the ecological requirements of the species where its invasion should be prioritized), moderately suitable (indicating that the environmental conditions in these areas partially meet the ecological requirements of the species, but there are significant factors that limit the survival of the species, and they may become temporary refuges for the species under climate change), low suitability (indicating that the environmental conditions in these areas only barely meet the critical conditions of the ecological requirements of the species, but they cannot sustain the population in the long term), and unsuitable. Under climate warming or human intervention, low-suitability areas may gradually transform into moderately or highly suitable areas. In this study, the suitable habitat areas were divided into four levels according to the rules of unsuitable areas (<0.05), low-suitability areas (0.05 ≤ x < 0.3), moderately suitable areas (0.3 ≤ x < 0.6), and highly suitable areas (≥0.6).

### 2.4. Extraction of Potential Suitability Distribution in Corn-Planting Range

Using ArcGIS 10.8, the current predicted distribution range of *D. virgifera virgifera*, *D. undecimpunctata*, and *D. barberi* in their suitable habitats were intersected with the actual national corn-planting range map of China in 2023 (as shown in Figure 2) to determine the areas with invasion risks. First, the predicted range map of the suitable habitats in China was resampled to 30 m, and the coordinate system was converted to “Albers_Conic_Equal_Area” to match the corn data. Then, using “Extract by Mask”, the three species’ suitability values for these *Diabrotica* beetles were extracted and exported. Finally, the suitable area maps of these three species of *Diabrotica* beetles in the corn-planting regions of China were drawn for subsequent analysis of the potential geographic distribution and occurrence areas of these *Diabrotica* beetles in the corn-planting regions of China.

## 3. Results

### 3.1. Model Performance

After combining RM and FC using the R package ENMeval and making separate predictions, three corresponding optimal model parameters were selected (ΔAICc = 0). Therefore, the optimal model settings for *D. barberi* and *D. virgifera virgifera* were both RM = 0.5 and FC = LHP, the RM for *D. undecimpunctata* was 0.5, and the FC was LQHPT (Table S1).

### 3.2. Response and Contributions of Three Diabrotica Species to Environmental Variables

**Figure 3:**
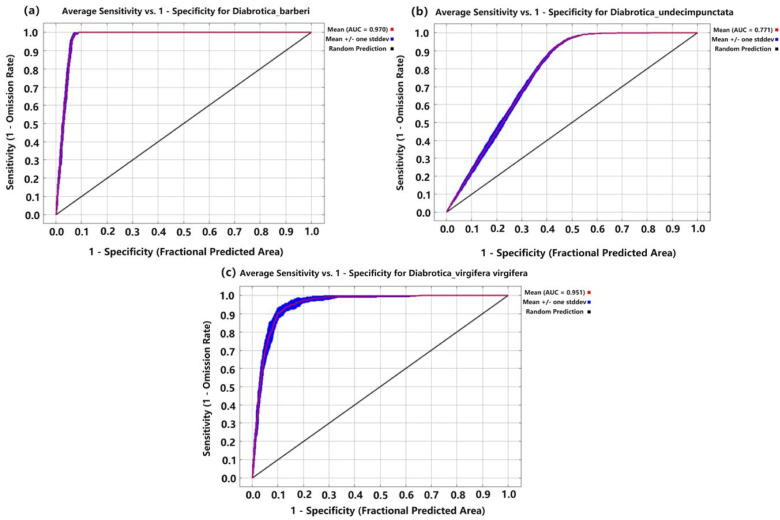
The AUC-ROC curves of the habitat suitability models for the three species of *Diabrotica* beetles based on MaxEnt (note: (**a**) *D. barberi*. (**b**) *D. undecimpunctata*. (**c**) *D. virgifera virgifera*).

As shown in Figure 3, the average training and test AUC value of *D.barberi* was both 0.970, and that of TSS was 0.9777; the average training AUC value of *D.undecimpunctata* was 0.773, its average test AUC value was 0.771, and that of TSS was 0.8568; the average training AUC value of *D. virgifera virgifera* was 0.955, its average test AUC value was 0.952, and that of TSS was 0.8583. This indicates that the optimized model showed high predictive ability and accuracy for these three species of *Diabrotica* beetles.

**Figure 4:**
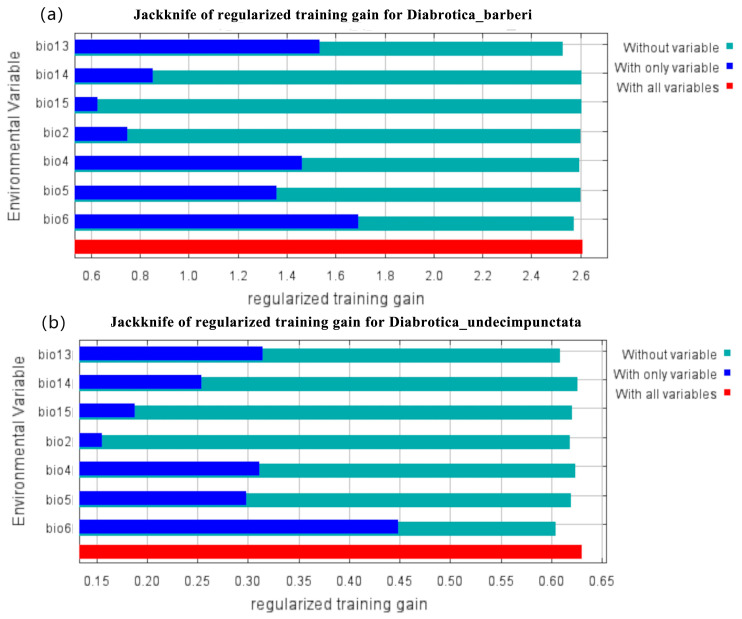

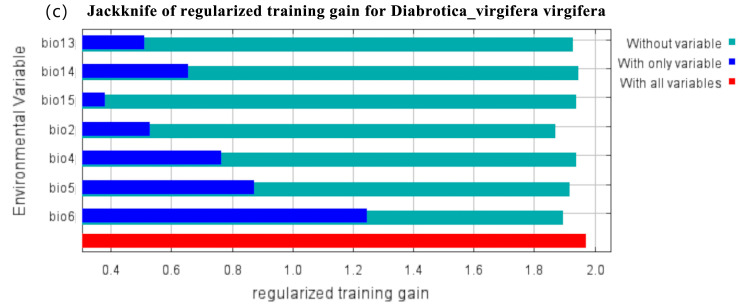
The variable importance determined by the Jackknife test for three *Diabrotica* species (note: (**a**) *D. barberi*. (**b**) *D. undecimpunctata*. (**c**) *D. virgifera virgifera*).

Furthermore, the Jackknife test was applied to determine the percentage contribution and ranking importance of each environmental variable to the three *Diabrotica* species in order to assess their relative importance (Figure 4). The results shown in Table 2 showed that the most important bioclimatic variables for *D. barberi* were bio4 (28.8%), bio13 (27.9%), bio6 (20.1%), and bio14 (16.0%), with a cumulative contribution rate of 92.7%; those for *D. undecimpunctata* were bio6 (55.7%), bio13 (19.0%), bio5 (10.5%), and bio14 (6.2%), with a cumulative contribution rate of 91.4%; and those for *D. virgifera virgifera* were bio6 (46.0%), bio14 (22.7%), bio5 (12.1%), and bio2 (10.2%), with a cumulative contribution rate of 91.0%. These environmental factors were identified as the main bioclimatic factors determining the suitable habitats of the three *Diabrotica* species (Table 2). Finally, six bioclimatic variables, namely, bio2, bio4, bio5, bio6, bio13, and bio14, were selected for the following analysis of the potential geographic distribution and occurrence areas of *D. virgifera virgifera*, *D. undecimpunctata*, and *D. barberi*.

### 3.3. Current Potential Geographic Distribution of the Three Diabrotica Species in China

**Figure 5:**
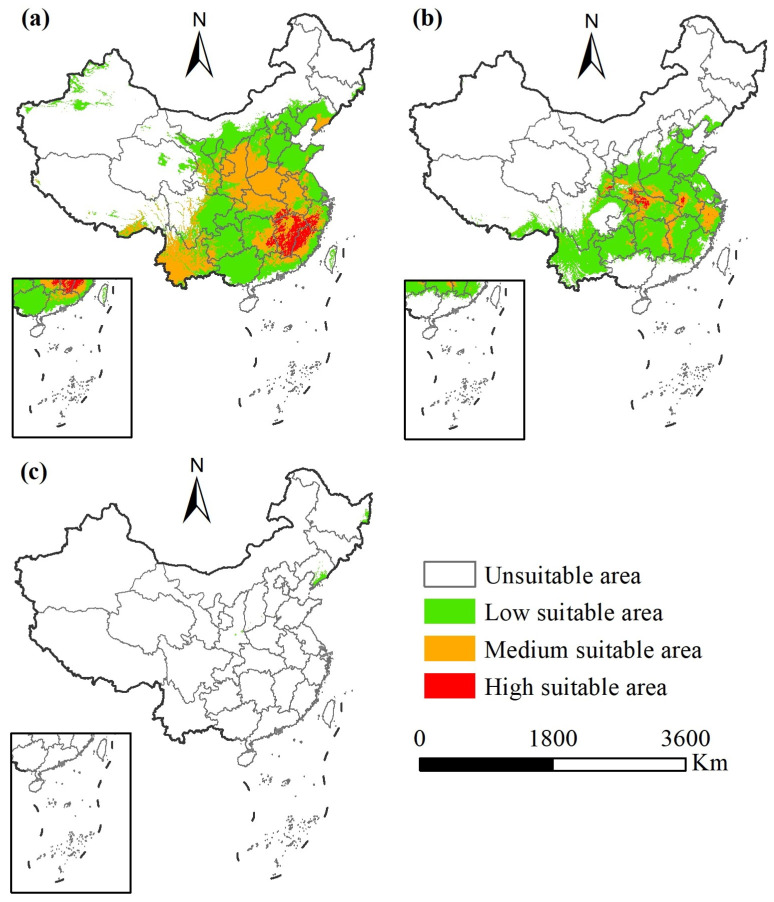
Potential geographic distribution of the three species of *Diabrotica* beetles in China at present (note: (**a**) *D. undecimpunctata*. (**b**) *D. virgifera virgifera*. (**c**) *D. barberi*).

The potential geographic distribution and suitable areas of the three species of *Diabrotica* beetles under the current climate conditions in China are shown in Figure 5 and Table 3. The suitable area ranges of *D. undecimpunctata* and *D. virgifera virgifera* are relatively large, exceeding one quarter of China’s land area, while *D. barberi* occupies only a small area in the provinces of Liaoning and Heilongjiang (Figure 5). Specifically, the potential total suitable area of *D. undecimpunctata* is 3.9332 × 10^8^ ha, accounting for approximately 41.0% of China’s land area, of which 2.2414 × 10^8^ ha (23.3%) is low-suitability area, 1.5107 × 10^8^ ha (15.8%) is moderately suitable areas, and 1.8116 × 10^7^ ha (1.9%) is high-suitability area (Table 3). The potential total suitable area of *D. virgifera virgifera* is 2.5061 × 10^8^ ha, accounting for approximately 26.1% of China’s land area, of which 2.1354 × 10^8^ ha (22.2%) is low-suitability area, 3.4097 × 10^7^ ha (3.6%) is moderately suitable area, and 2.973 × 10^6^ ha (0.3%) is high-suitability areas (Table 3). *D. barberi* is in all low-suitability areas, with only 2.877 × 10^6^ ha, accounting for approximately 0.3% of China’s land area (Table 3).

The potential geographic distribution range of *D. undecimpunctata* is approximately 21.01–48.46° N, 74.01–131.26° E, covering the central, eastern, and southern parts of China, and the main areas where it is expected to be distributed include the provinces of Zhejiang, Jiangxi, Fujian, Guangdong, Hunan, Hubei, Anhui, Henan, Shanxi, Shaanxi, and Yunnan, etc. (Figure 5a). The potential geographic distribution range of *D. virgifera virgifera* is approximately 21.58–41.42° N, 78.71–124.43° E, covering the central, eastern, and southwestern regions of China, and the main areas where it is expected to be distributed include the province of Zhejiang, Anhui, western Jiangxi, eastern Hunan, northwestern Hubei, southern Shanxi, southern Henan, etc. (Figure 5b). The potential geographic distribution range of *D. barberi* is approximately 34.21–46.81° N, 108.80–133.75° E, and it is expected to have sporadic distribution only in the southern part of Liaoning province and the northeastern part of Heilongjiang province, with limited distribution (Figure 5c).

### 3.4. Overlay Analysis of the Current Potential Geographic Distribution of the Three Diabrotica Species and the Actual Distribution of Corn-Growing Regions in China

Based on the overlay analysis of the current potential geographic distribution of the three *Diabrotica* species (Figure 5) and the actual distribution of corn-growing regions in China (Figure 2), the potential geographic distribution area of *D. undecimpunctata*, *D. virgifera virgifera*, and *D. barberi* on corn crops in China were calculated, and the results are shown in Table 4. *D. undecimpunctata* has the largest potential geographic distribution area in 22 provinces, totaling approximately 2.618 × 10^7^ ha (accounting for about 59.21% of the corn-planting area, including 6.08 × 10^4^ ha of highly suitable area, 1.0937 × 10^7^ ha of medium-suitability area, and 1.5182 × 10^7^ ha of low-suitability area), mainly distributing in the provinces of Shandong, Henan, Hubei, Liaoning, Sichuan, and Shanxi (Table 4). Table 4 also indicates that the highly suitable area of *D. undecimpunctata* is distributed in the provinces of Hunan (3.04 × 10^4^ ha), Hubei (2.81 × 10^4^ ha), Anhui (0.18 × 10^4^ ha), and Gansu (0.04 × 10^4^ ha).

D. virgifera virgifera also has a large potential geographic distribution area covering 20 provinces, totaling approximately 1.8141 × 10^7^ ha (accounting for about 41.03% of the corn-planting area, including 5.26 × 10^4^ ha of highly suitable area, 1.2434 × 10^6^ ha of medium-suitability area, and 1.6845 × 10^7^ ha of low-suitability area), mainly distributing in the provinces of Shandong, Henan, Hubei, Anhui, and Yunnan (Table 4). Table 4 also indicates that the highly suitable area of *D. virgifera virgifera* is distributed in the provinces of Shaanxi (2.54 × 10^4^ ha), Hubei (2.01 × 10^4^ ha), Gansu (0.36 × 10^4^ ha), Chongqing (0.18 × 10^4^ ha), Anhui (0.16 × 10^4^ ha), Hunan (0.02 × 10^4^ ha), and (0.01 × 10^4^ ha). Meanwhile, *D. barberi* has the lowest potential geographic distribution area, covering only eight provinces, totaling approximately 44.37 × 10^4^ ha (accounting for about 1.01% of the corn-planting area, with this being only low-suitability area), mainly distributed in the provinces of Heilongjiang and Liaoning (Table 4).

### 3.5. Changes in Potential Geographic Distribution Range of the Three Diabrotica Species in China in the Future

**Figure 6:**
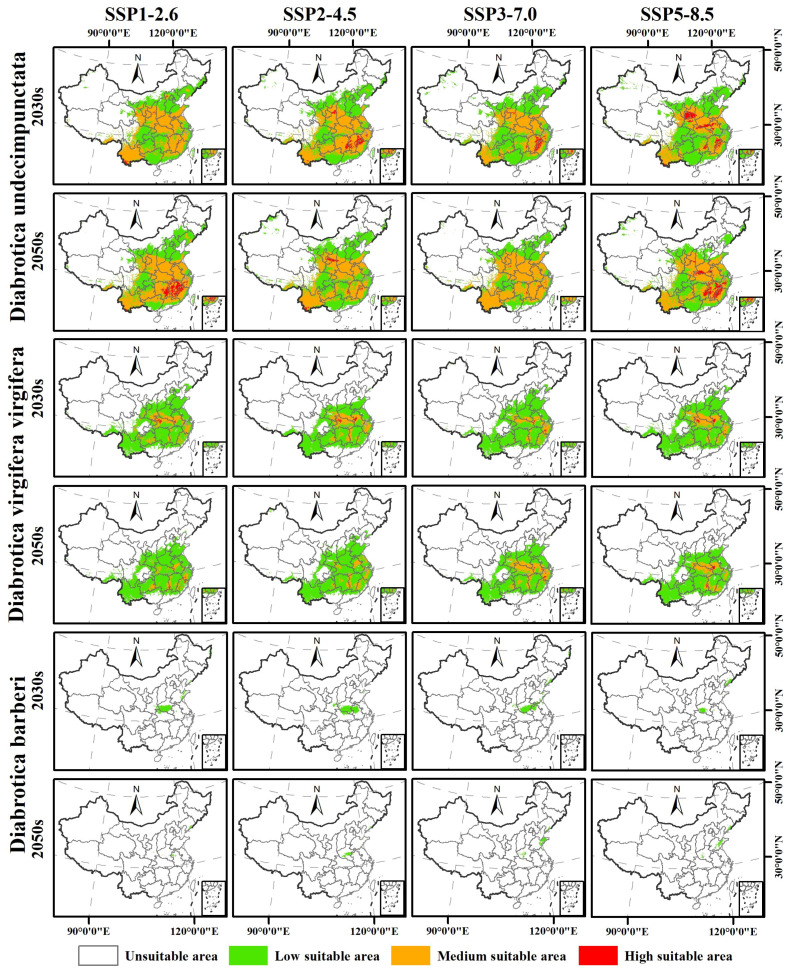
The suitable area ranges of the three species of *Diabrotica* beetles, *D. undecimpunctata*, *D. virgifera virgifera*, and *D. barberi*, under different climate scenarios in different future periods.

**Figure 7:**
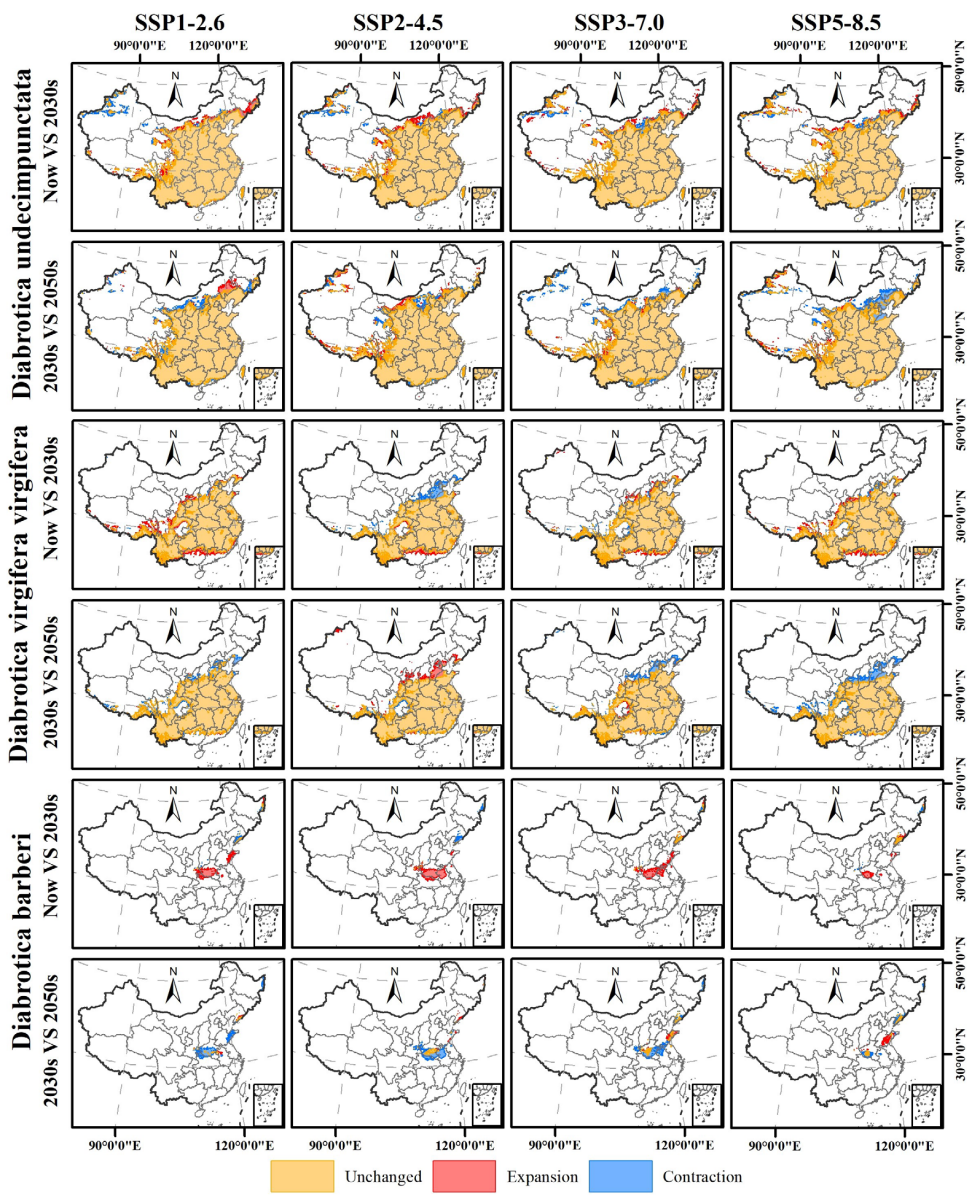
Potential geographic distribution changes in the potential suitable area of three *Diabrotica* species, *D. undecimpunctata*, *D. virgifera virgifera*, and *D. barberi*, under different climate scenarios in different future periods (Note: “Unchanged” indicates ranges that remained unchanged between the two periods; “Expansion” represents ranges that exceeded the previous period in the latter period; and “Contraction” shows ranges that did not exist in the previous period but existed in the latter period).

For these *Diabrotica* beetles, *D. undecimpunctata* and *D. virgifera virgifera* occupy relatively large potentially suitable areas in China under the four climate scenarios of SSP1_2.6, SSP2_4.5, SSP3_7.0, and SSP5_8.5 for the 2030s and 2050s (Figure 6), and they have only sporadic increases or decreases surrounding their potentially suitable area ranges under the current scenarios (Figure 7). For *D. barberi*, its suitable area expanded under the four climate scenarios for the 2030s except under SSP5_8.5 (Figure 6; Table 5). Specifically, the suitable area of *D. undecimpunctata* did not change under the four climate scenarios for the 2030s and 2050s, with only a decrease of less than 2%, but it is worth noting that its highly suitable area decreased, while its medium- and low-suitability areas increased accordingly (Figure 6; Table 5).

For *D. virgifera virgifera*, its suitable area in the SSP1_2.6 scenario for the 2030s relative to the current climate expanded by approximately 11%, and that in the SSP5_8.5 scenario for the 2030s expanded by approximately 7% (Table 5). Moreover, its suitable area in the SSP1_2.6 scenario for the 2050s relative to the 2030s decreased by approximately 8%, and that in the SSP5_8.5 scenario for the 2050s decreased by approximately 12% (Table 5). Furthermore, it is worth noting that regardless of the period, its highly suitable area also decreased, while its medium- and low-suitability areas increased (Table 5).

For *D. barberi*, compared with the current period, its suitable area in the four climate scenarios for the 2030s showed significant expansion, with the largest increase in the SSP3_7.0 scenario, increasing by approximately 426% (Table 5). However, its suitable area in the four climate scenarios for the 2050s relative to the 2030s showed a significant reduction, with the largest reduction in the SSP1_2.6 scenario, reducing by approximately 86% (Table 5).

In addition, the suitable area changes of *D. undecimpunctata* under the different climate scenarios for the 2030s and 2050s indicate that it will sporadically appear in regions such as Tibet, Sichuan, Xinjiang, Inner Mongolia, Liaoning, Jilin, and Heilongjiang, with relatively small area changes (Figure 7). The suitable area of *D. virgifera virgifera* will expand in Guangdong, Guangxi, Tibet, Sichuan, Ningxia, and Shaanxi in the 2030s, except for under scenario SSP2_4.5, and its suitable area in the other climate scenarios, except for SSP2_4.5, will reduce in Liaoning, Beijing, Tianjin, Hebei, Shanxi, Shaanxi, Sichuan, and Tibet (Figure 7). Moreover, the suitable area of *D. barberi* will expand under the different climate scenarios for the 2030s, with the main expansion areas in Hubei, Shaanxi, Henan, Anhui, and Shandong, and the expanded areas in the 2050s relative to the 2030s will show a partial reduction trend (Figure 7).

### 3.6. Analysis of the Response of Dominant Environmental Variables to the Potential Geographic Distribution Probabilities of the Diabrotica Beetles in the MaxEnt Model

**Figure 8:**
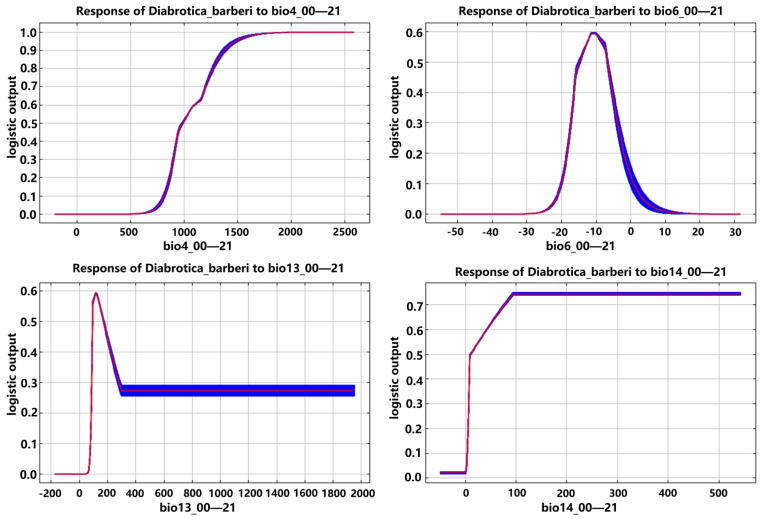
Response curves of *D. barberi* to the four main environmental variables (note: The blue area represents the range of occurrence probabilities, while the red curve indicates the average occurrence probability. bio4—temperature seasonality; bio6—min temperature of the coldest month; bio13—precipitation in the wettest month; bio14—precipitation in the driest month).

**Figure 9:**
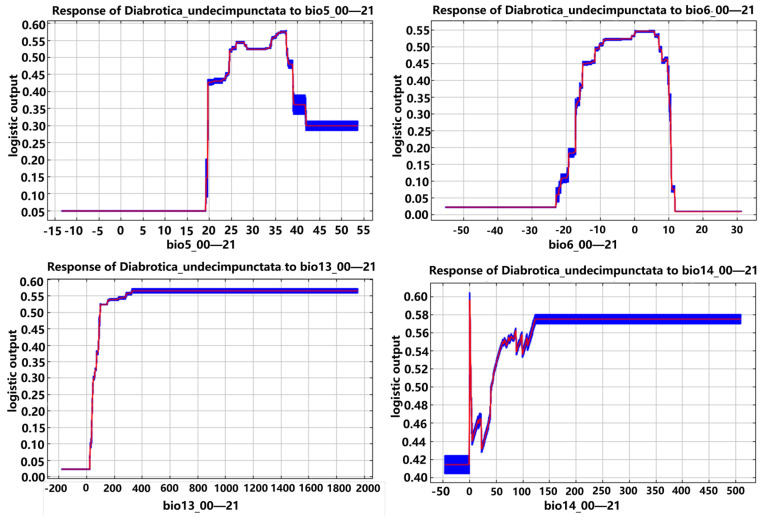
Response curves of *D. undecimpunctata* to the four main environmental variables (note: The blue area represents the range of occurrence probabilities, while the red curve indicates the average occurrence probability. bio5—max temperature of the warmest month; bio6—min temperature of the coldest month; bio13—precipitation in the wettest month; bio14—precipitation in the driest month).

**Figure 10:**
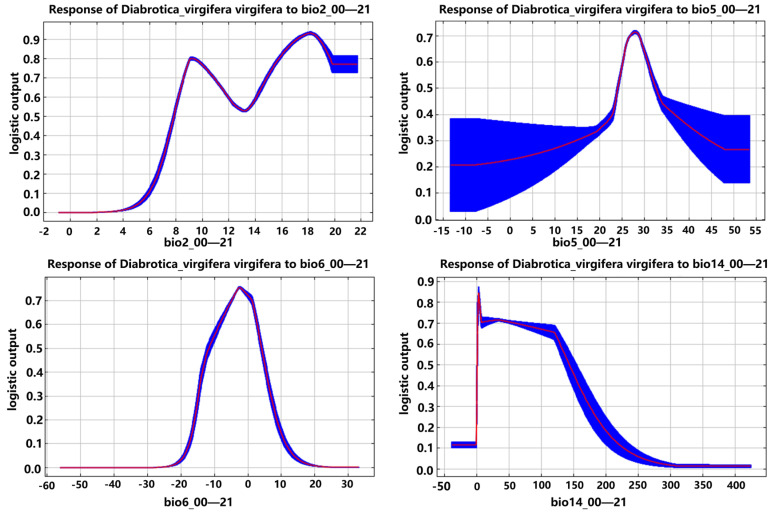
Response curves of *D. virgifera virgifera* to four major environmental variables (note: The blue area represents the range of occurrence probabilities, while the red curve indicates the average occurrence probability. bio2—mean diurnal range; bio5—max temperature of the warmest month; bio6—min temperature of coldest month; bio14—precipitation in the driest month).

Based on the response curves of environmental variables to the distribution probabilities of *D. barberi*, *D. undecimpunctata*, and *D. virgifera virgifera* in the MaxEnt model, the main environmental variable ranges suitable for their potential geographic distribution were determined (Figure 8, Figure 9 and Figure 10; Table 6). The suitable ranges of environmental variables for *D. barberi* were bio4 (985.5–2589.1, with an optimal value of 1966.7), bio6 (−14.9–−6.8 °C, with an optimal value of −11.1 °C), bio13 (95.1–167.5 mm, with an optimal value of 116.4 mm), and bio14 (9.9–542.9 mm, with an optimal value of 95.2 mm) (Table 6). After reaching the optimal value of bio6, the distribution probability decreased as its value increased, and *D. barberi* maintained a relatively high distribution probability after reaching the maximum values of bio4, bio13, and bio14, regardless of any changes in them (Figure 8).

The suitable ranges for *D. undecimpunctata* were bio5 (24.7–37.6 °C, with an optimal value of 35.6 °C), bio6 (−10.3–7.8 °C, with an optimal value of 4.4 °C), bio13 (99.3–1951.3 mm, with an optimal value of 286.7 mm), and bio14 (42.6–509.1 mm, with an optimal value of 124.8 mm) (Table 6). After reaching the maximum value of bio6, its distribution probability decreased as its value increased, and it maintained a relatively high distribution probability after reaching the maximum value of bio5, bio13, and bio14, regardless of any changes in them (Figure 9).

The suitable ranges for *D. virgifera virgifera* were bio2 (7.8–21.7 °C, with an optimal value of 18.2 °C), bio5 (24.4–32.7 °C, with an optimal value of 27.8 °C), bio6 (−12.4–3.7 °C, with an optimal value of −2.6 °C), and bio14 (1.2–156.9 mm, with an optimal value of 3.1 mm) (Table 6). After reaching the maximum values of bio6 and bio14, its distribution probability decreased as its value increased, and it maintained a relatively high distribution probability after reaching the maximum values of bio2 and bio5, regardless of any changes in them (Figure 10).

## 4. Discussion

*Diabrotica* beetles, including the western corn rootworm *D. virgifera virgifera*, the southern corn rootworm *D. undecimpunctata*, and the northern corn rootworm *D. barberi*, are important insect pests of corn crops that natively occur in Central America [1,5,25]. *D. virgifera virgifera* and *D. barberi* are univoltine species; they generally overwinter as eggs [1]. *D. undecimpunctata* is a multivoltine species that often overwinters as adults in the south and east of the Midwestern United States, and their offsprings reinvade the USA “Corn Belt” each year [26]. In addition, these three *Diabrotica* beetles can co-occur in the northeastern United States. The eggs of *D. virgifera virgifera* and *D. barberi* hatch and thrive in the late spring, while *D. undecimpunctata* is abundant in the winter [1]. Both *D. virgifera virgifera* and *D. barberi* exhibit a strong preference for corn, whereas *D. undecimpunctata* does not show a strong preference for corn when there are other high-quality foods available in the northeastern United States [27]. Before 1955, *D. virgifera virgifera* was just distributed in the Midwestern regions of America, and it expanded rapidly eastward with a diffusion rate of 60–80 km over the next 50 years, after which it spread to the Atlantic coast from 1985 to 2005 [28]. In 1992, *D. virgifera virgifera* first occurred in a corn field near the airport in Belgrade and then spread rapidly to many countries in Europe [29]. Adults of *D. virgifera virgifera* have strong flight capability, enabling them to migrate over long distances. Moreover, the primary route for the introduction of *D. virgifera virgifera* to Europe is through human activities, particularly air transportation [30]. The extensive cornfields in Europe coupled with the absence of natural predators have facilitated the rapid proliferation and spread of *D. virgifera virgifera*. To date, there have been no reports of *D. barberi* and *D. undecimpunctata* invading Europe. However, these two *Diabrotica* beetles have been included in regulation (EU) 2016/2031, and they both meet the criteria assessed by EFSA for species to be considered a potential European Union quarantine pest [31,32].

In North America, *D. virgifera virgifera* annually costs 800 million USD of corn yield losses and 200 million USD losses from pest control fees, totaling 1 billion USD lost, leading to it being named as a “1 billion-dollar pest” [6,7]. In addition, in America, *D. virgifera virgifera* is mainly distributed between 35–49° N (as shown in Figure 1), and about 70% of the total corn yield is harvested from this corn-planting region of America [33]. Corn is also one of the main crops in China, where its planting area is mainly distributed between 30–50° N (as shown in Figure 2), and these geographical latitudes and climate types are similar to those of North America. Once *D. virgifera virgifera* is introduced into China, it is very likely to spread, settle, and develop rapidly. To date, *D. virgifera virgifera* has not been detected in China, although there is invasion risk because it can be transmitted by human activity, and its adults have a strong flying ability [8]. Early in 2002, Zhang and Yang [33] warned of the invasion of the hazard pest *D. virgifera virgifera* in China.

Species distribution models (SDMs) have good predictive performance in evaluating suitable areas for the establishment of global invasive species [16,17]. In 2024, Jin et al. [8] used eight algorithms (including FDA, GAM, CTA, RF, GLM, MARS, GBM, and MaxEnt) to estimate the global geographical distribution patterns and ecological niche overlap of three species of corn rootworms, *D. virgifera virgifera*, *D. undecimpunctata*, and *D. barberi*, although they did not consider the actual distribution of the host corn crop, which is a necessary condition for its successful colonization and damage. In this study, based on the relative importance of each environmental variable to the three species of *Diabrotica* beetles, as determined by the Jackknife test, the most important bioclimatic variables for *D. barberi* were bio4, bio13, bio6, and bio14, with a cumulative contribution rate of 92.7%; those for *D. undecimpunctata* were bio6, bio13, bio5, and bio14, with a cumulative contribution rate of 91.4%; and those for *D. virgifera virgifera* were bio6, bio14, bio5, and bio2, with a cumulative contribution rate of 91.0%. So, these six environmental factors (i.e., bio2, bio4, bio5, bio6, bio13, and bio14) were identified as the main bioclimatic factors determining the potential suitable habitats of the three *Diabrotica* species. Jin et al. [8] also used these climate variables for predicting the suitable areas of *D. virgifera virgifera*, *D. undecimpunctata*, and *D. barberi*.

In 2009, Zhang et al. [34] utilized the GRAP software (Version: 1.1.6) to predict their potential geographic distribution in China, and they indicated that *D. virgifera virgifera* could establish itself in the southern Northeastern Plain, the southern North China Plain, and the Weihe Plain, overlapping the main corn-planting regions in China. In this study, the suitable area ranges of *D. undecimpunctata* and *D. virgifera virgifera* are relatively large in China, and the potential geographic distribution range is 21.01–48.46° N and 74.01–131.26° E for *D. undecimpunctata* and 21.58–41.42° N and 78.71–124.43° E for *D. virgifera virgifera*, respectively, while *D. barberi* occupies only a small area in China, and its potential geographic distribution range is 34.21–46.81° N and 108.80–133.75° E. Based on the overlay analysis of the current potential geographic distribution of the three *Diabrotica* species and the actual distribution of corn-growing regions in China, *D. undecimpunctata* and *D. virgifera virgifera* have the largest potential geographic distribution areas, totaling approximately 2.618 × 10^7^ ha and 1.8141 × 10^7^ ha in 22 and 20 provinces, respectively, while *D. barberi* has the lowest potential geographic distribution area in just 8 provinces, totaling 44.37 × 10^4^ ha of low-suitability area, mainly in the provinces of Liaoning and Heilongjiang. Jin et al. [8] indicated that the potential geographic distribution area of *D. virgifera virgifera* will expand further and reach a maximum under the SSP5-8.5 scenario in the 2050s (globally 2499 × 10^4^ km^2^), it has the highest potential for invasion under the current and future global warming scenarios, and the degree of ecological niche overlap was the highest for *D. undecimpunctata* and *D. virgifera virgifera*, with the highest similarity in the potential geographic distribution pattern and maximum coexistence range.

In this study, under the four climate scenarios (i.e., SSP1_2.6, SSP2_4.5, SSP3_7.0, and SSP5_8.5) for the 2030s and 2050s, these *Diabrotica* beetles only have sporadic increases or decreases in the surrounding areas of the current period’s suitable area range, and it is worth noting that the highly suitable areas of *D. undecimpunctata* and *D. virgifera virgifera* decreased, while their medium- and low-suitability areas increased accordingly. So, it is presumed that the *Diabrotica* beetles, especially *D. virgifera virgifera* and *D. undecimpunctata*, have a high risk of potential invasion into China because there are large potentially suitable areas for their possible occurrence in the corn-planting regions of China. In this study, the temperature factor was primarily taken into consideration. However, there are many factors that influence the distribution of *Diabrotica* beetles. Soil properties usually affect their oviposition preferences, which, in turn, influences their distribution, e.g., *D. undecimpunctata* prefers moist soil and dark soil with moderate organic matter and clay content for oviposition [35]. In addition, host plants are also a major factor influencing the distribution of these *Diabrotica* beetles. Larvae of both *D. virgifera virgifera* and *D. barberi* are oligophagous, and their host range is limited to corn, although they can sustain a low population level on a few Poaceae hosts in the absence of corn [1,36]. Some studies have indicated that *D. virgifera virgifera* adults exhibit greater longevity when feeding on corn compared to feeding on other host plants [37]. Thus, the distribution ranges of *D. virgifera virgifera* and *D. barberi* are closely tied to the distribution range of their primary host plant, corn. In contrast, *D. undecimpunctata* is polyphagous, and the larvae feed on a variety of host plants, including Asteraceae, Chenopodiaceae, Cucurbitaceae, Fabaceae, Poaceae, Polygonaceae, and Solanaceae [38]. *D. undecimpunctata* is primarily distributed in North and Central America, and in these regions, major crops (e.g., soybeans, cotton, and sweet potatoes) can also serve as foods for *D. undecimpunctata*. Therefore, we need to pay more attention to other host plants that influence the distribution of *D. undecimpunctata* in future studies.

This study has some limitations. Although the MaxEnt model provides valuable predictions, the current analysis is mainly limited to differences in climate ecological niches and does not consider environmental factors closely related to the growth of pests, such as soil moisture and soil temperature. Moreover, although we collected all the data on the occurrence of *Diabrotica* beetles as much as possible, we have to admit that there are still some prediction biases caused by the limited occurrence data. Therefore, future research is necessary to incorporate various types of environmental variables (such as climatic factors, soil characteristics, etc.) and integrate complete occurrence data as much as possible to generate more accurate habitat predictions and provide a scientific basis for formulating effective pest invasion control strategies.

## 5. Conclusions

The three species of *Diabrotica* beetles studied here, including *D. virgifera virgifera*, *D. undecimpunctata*, and *D. barberi*, have a risk of invasion because they can be naturally or anthropogenically transmitted into China. In this study, the results indicated that six bioclimatic variables (i.e., bio2, bio4, bio5, bio6, bio13, and bio14) were selected for the analysis of the potential geographic distribution and suitable areas of these *Diabrotica* beetles. The suitable area ranges of *D. undecimpunctata* and *D. virgifera virgifera* are relatively large in China, while *D. barberi* occupies only a small area in China. Simultaneously, *D. undecimpunctata* and *D. virgifera virgifera* have the largest potential geographic distribution areas in 22 and 20 provinces, respectively, while *D. barberi* has the lowest potential geographic distribution area in just 8 provinces. Moreover, these *Diabrotica* beetles only have sporadic increases or decreases surrounding their potential suitable areas under the four climate scenarios (i.e., SSP1_2.6, SSP2_4.5, SSP3_7.0, and SSP5_8.5) in the 2030s and 2050. However, it is worth noting that the highly suitable areas of *D. undecimpunctata* and *D. virgifera virgifera* decreased, and their medium- and low-suitability areas increased accordingly. It is presumed that the *Diabrotica* beetles, especially *D. virgifera virgifera* and *D. undecimpunctata*, have a high potential risk of invasion into China because there are large potential suitable distribution areas for their possible occurrence in the maize-planting regions of China.

## Figures and Tables

**Table 1:** The bioclimatic variables and their description.

Variables	Description
bio2 (°C)	Mean diurnal range (mean of monthly max temp—min temp)
bio4	Temperature seasonality (standard deviation × 100)
bio5 (°C)	Max temperature of the warmest month
bio6 (°C)	Min temperature of coldest month
bio13 (mm)	Precipitation of wettest month
bio14 (mm)	Precipitation of driest month
bio15 (mm)	Precipitation seasonality (coefficient of variation)

**Table 2:** The percentage contribution and ranking of environmental variables in the MaxEnt model of *Diabrotica barberi*, *D. undecimpunctata*, and *D. virgifera virgifera*.

Code	*D. barberi*		*D. undecimpunctata*		*D. virgifera virgifera*	
	Percentage Contribution (%)	Permutation Importance (%)	Percentage Contribution (%)	Permutation Importance (%)	Percentage Contribution (%)	Permutation Importance (%)
bio2	0.6	1.4	3.3	6.2	10.2	7.7
bio4	28.8	32.4	3.3	6.1	1.6	5.7
bio5	6.0	7.7	10.5	11.6	12.1	3.4
bio6	20.1	26.5	55.7	45.5	46.0	58.6
bio13	27.9	27.8	19.0	20.5	5.9	12.3
bio14	16.0	2.9	6.2	4.6	22.7	5.3
bio15	0.6	1.3	2.0	5.5	1.5	7.0

Note: The bioclimatic variables and their description of bio2, bio4, bio5, bio6, bio13, bio14, and bio15 are shown in Table 1.

**Table 3:** Potential suitable area of the three species of *Diabrotica* beetles in China at the present period.

	Highly Suitable Area (×10^4^ ha)	Moderately Suitable Area (×10^4^ ha)	Low Suitable Area (×10^4^ ha)	Total Suitable Area (×10^4^ ha)
*D. barberi*	0	0	287.7	287.7
*D. virgifera virgifera*	297.3	3409.7	21,354.2	25,061.1
*D. undecimpunctata*	1811.6	15,106.7	22,413.5	39,331.8

**Table 4:** Potential suitable area of the three *Diabrotica* species on corn crops in China based on the overlay analysis of their potential geographic distribution and the actual distribution of Chinese corn-growing regions in 2023.

Province	*D. barberi*			*D. undecimpunctata*			*D. virgifera virgifera*		
	H-Area (×10^4^ ha)	M-Area (×10^4^ ha)	L-Area (×10^4^ ha)	H-Area (×10^4^ ha)	M-Area (×10^4^ ha)	L-Area (×10^4^ ha)	H-Area (×10^4^ ha)	M-Area (×10^4^ ha)	L-Area (×10^4^ ha)
AH	/	/	/	0.18	110.61	1.71	0.16	3.06	109.24
CQ	/	/	/	/	2.88	41.36	0.18	1.47	9.59
GS	/	/	/	0.04	45.46	43.74	0.36	3.41	38.36
GX	/	/	/	/	2.73	50.61	/	/	6.10
GZ	/	/	/	/	19.47	40.74	/	1.88	54.88
HEB	/	/	0.53	/	6.30	325.11	/	/	263.88
HLJ	/	/	21.75	/	/	1.54	/	/	/
HEN	/	/	0.06	/	359.61	20.44	/	38.89	341.15
HUB	/	/	/	2.81	52.39	22.05	2.01	41.05	34.19
HUN	/	/	/	3.04	18.31	14.58	0.02	3.37	31.91
Inner	/	/	/	/	1.93	85.22	/	/	/
JL	/	/	0.24	/	0.71	6.17	/	/	0.88
JS	/	/	/	/	28.83	22.59	/	0.03	49.22
LN	/	/	19.51	/	30.25	197.82	/	/	32.18
NX	/	/	/	/	8.05	23.02	/	/	4.24
SAX	/	/	1.18	/	110.15	9.50	2.54	28.55	60.71
SC	/	/	/	/	10.96	174.28	0.01	1.07	43.71
SD	/	/	0.03	/	92.02	301.24	/	/	382.48
SX	/	/	1.09	/	112.75	64.08	/	/	98.62
TJ	/	/	/	/	/	18.64	/	/	18.55
XJ	/	/	/	/	/	12.15	/	/	0.01
YN	/	/	/	/	80.30	41.63	/	1.57	104.59
Total	/	/	44.37	6.08	1093.71	1518.23	5.26	124.34	1684.49

Note: /—no existing; H-area—high-suitability area; M-area—medium-suitability area; L-area—low-suitability area. AH—Anhui; CQ—Chongqing; GS—Gansu; GX—Guangxi; GZ—Guizhou; HEB—Hebei; HLJ—Heilongjiang; HEN—Henan; HUB—Hubei; HUN—Hunan; Inner—Inner Mongolia; JL—Jilin; JS—Jiangsu; LN—Liaoning; NX—Ningxia; SAX—Shaanxi; SC—Sichuan; SD—Shandong; SX—Shanxi; TJ—Tianjin; XJ—Xinjiang; YN—Yunnan.

**Table 5:** Potential suitable area (×104 ha) of the three *Diabrotica* species, *D. barberi*, *D. undecimpunctata*, and *D. virgifera virgifera* in China under different climate scenarios for the 2030s and 2050s.

Species	Suitable Area	2030s				2050s			
		SSP1_2.6	SSP2_4.5	SSP3_7.0	SSP5_8.5	SSP1_2.6	SSP2_4.5	SSP3_7.0	SSP5_8.5
*D. b*	H-area	/	/	/	/	/	/	/	/
	M-area	/	/	/	/	/	/	/	/
	L-area	1367.7	1479.2	1512.0	623.3	191.9	374.1	539.3	446.1
*D. u*	H-area	247.1	1011.1	700.8	1778.3	1732.1	486.3	122.0	1718.2
	M-area	19,988.5	19,370.6	15,723.4	13,689.5	19,702.9	18,264.2	22,848.6	17,130.7
	L-area	19,760.9	19,475.0	22,502.1	23,554.9	17,251.8	21,356.6	14,574.6	17,364.8
	Total-area	39,996.5	39,856.7	38,926.3	39,022.7	38,686.8	40,107.2	37,545.2	36,213.7
*D. v*	H-area	168.3	210.5	88.2	189.0	116.2	5.1	110.1	142.8
	M-area	4577.5	4877.3	3276.1	4469.5	2587.5	2902.1	4880.7	3462.3
	L-area	23,083.2	19,897.7	22,497.0	22,166.2	22,883.4	22,923.8	19,810.3	19,898.4
	Total-area	27,829.0	24,985.5	25,861.3	26,824.7	25,587.1	25,831.0	24,801.1	23,503.5

Note: *D. b*—*D. barberi*; *D. u*—*D. undecimpunctata*; *D. v*—*D. virgifera virgifera*; /—no existing; H-area—high-suitability area; M-area—medium-suitability area; L-area—low-suitability area; Total-area—total suitable area.

**Table 6:** Appropriate range and optimum value of the suitable environmental variables for the *Diabrotica* beetles, *D. barberi*, *D. undecimpunctata*, and *D. virgifera virgifera*.

Code	*D. barberi*		*D. undecimpunctata*		*D. virgifera virgifera*	
	Appropriate Range	Optimum	Appropriate Range	Optimum	Appropriate Range	Optimum
bio2	/	/	/	/	7.8~21.7 °C	18.2 °C
bio4	985.5~2589.1	1966.7	/	/	/	/
bio5	/	/	24.7~37.6 °C	35.6 °C	24.4~32.7 °C	27.8 °C
bio6	−14.9~−6.8 °C	−11.1 °C	−10.3~7.8 °C	4.4 °C	−12.4~3.7 °C	−2.6 °C
bio13	95.1~167.5 mm	116.4 mm	99.3~1951.3 mm	286.7 mm	/	/
bio14	9.9~542.9 mm	95.2 mm	42.6~509.1 mm	124.8 mm	1.2~156.9 mm	3.1 mm

Note: /—no existing; the bioclimatic variables bio2, bio4, bio5, bio6, bio13, and bio14 and their descriptions are shown in Table 1.

## Data Availability

The original contributions presented in this study are included in the article/Supplementary Materials. Further inquiries can be directed to the corresponding authors.

## Supplementary Materials

The following supporting information can be downloaded at https://www.mdpi.com/article/10.3390/insects16101072/s1, Table S1: Complete ENMeval tuning results for all parameter combinations. Dataset S1: Cleaned occurrence records with full metadata; Code S1: R scripts for data processing and analysis.
